# Spatial Variability of Picoeukaryotic Communities in the Mariana Trench

**DOI:** 10.1038/s41598-018-33790-4

**Published:** 2018-10-18

**Authors:** Hongmei Jing, Yue Zhang, Yingdong Li, Wenda Zhu, Hongbin Liu

**Affiliations:** 1 CAS Key Laboratory for Experimental Study under Deep-sea Extreme Conditions, Institute of Deep-sea Science and Engineering, Chinese Academy of Sciences, Sanya, China; 2 University of Chinese Academy of Sciences, Beijing, China; 3 Division of Life Science, The Hong Kong University of Science and Technology, Clear Water Bay, Kowloon, Hong Kong SAR, China

## Abstract

Picoeukaryotes play prominent roles in the biogeochemical cycles in marine ecosystems. However, their molecular diversity studies have been confined in marine surface waters or shallow coastal sediments. Here, we investigated the diversity and metabolic activity of picoeukaryotic communities at depths ranging from the surface to the abyssopelagic zone in the western Pacific Ocean above the north and south slopes of the Mariana Trench. This was achieved by amplifying and sequencing the V4 region of both 18S ribosomal DNA and cDNA using Illumina HiSeq sequencing. Our study revealed: (1) Four super-groups (i.e., Alveolata, Opisthokonta, Rhizaria and Stramenopiles) dominated the picoeukaryote assemblages through the water column, although they accounted for different proportions at DNA and cDNA levels. Our data expand the deep-sea assemblages from current bathypelagic to abyssopelagic zones. (2) Using the cDNA-DNA ratio as a proxy of relative metabolic activity, the highest activity for most subgroups was usually found in the mesopelagic zone; and (3) Population shift along the vertical scale was more prominent than that on the horizontal differences, which might be explained by the sharp physicochemical gradients along the water depths. Overall, our study provides a better understanding of the diversity and metabolic activity of picoeukaryotes in water columns of the deep ocean in response to varying environmental conditions.

## Introduction

Marine picoeukaryotes, (i.e., picoplanktonic eukaryotes of <2 μm in size), are capable of photosynthetic, heterotrophic and mixotrophic metabolisms^1^. Photosynthetic picoeukaryotes account for a large fraction of the biomass and the primary production in marine environments, they exhibit a remarkable high level of diversity in the surface waters, and they contribute a lot to the global carbon and mineral cycles^2–4^. Heterotrophic picoeukaryotes are the main bacterivorous grazers, and they integrate the microbial loop into classical marine food webs by transferring dissolved organic matter (DOM) utilized by heterotrophic bacteria to higher trophic levels^2^. Mixotrophic picoeukaryotes are also widespread in aquatic systems^5^, and these have been shown to play an ecologically significant role as primary producers and consumers^6^. Many picoeukaryotes are parasite, this is an efficient strategy that facilitates constant access to higher concentrations of organic materials^7^. Their various metabolic status together with high morphological and genetic diversity enables them to fulfill diverse roles in marine microbial ecosystems, especially in the biogeochemical cycles of carbon and nitrogen, as well as a number of other elements^7^.

Through next generation sequencing, novel picoeukaryotic assemblages and their ecological roles were revealed in various marine environments, including the Arctic^8^ and Pacific Oceans^9^, the Sargasso Sea^10^, as well as various European coastal sites^11^. Recently, an ocean microbial reference gene catalog (providing information about picoeukaryotes as well as other common microbiota) was established from data collected during global scientific voyages (Tara Oceans)^12^. However, most of the sequencing studies on picoeukaryotes are limited in euphotic/ disphotic zone^8–11^, and their diversities and ecological functions in the bathypelagic and abyssopelagic zones are only started to be studied in the recent years. For example, Pernice *et al*. investigated deep-sea microbial eukaryotic assemblages from 27 stations located in the Atlantic, Indian and Pacific oceans^13^, which is the first attempt to describe the global diversity of bathypelagic heterotrophic microbial eukaryotes by high-throughput sequencing.

Studies of picoeukaryotes at RNA level enable the identification of active components of the communities, which are different from those identified from DNA level studies^14,15^, because the latter contains moribund, encysted, metabolically inactive or even non-living genetic materials^16,17^. Thus comparative studies simultaneously at the DNA and RNA levels have been applied to marine protistans^3,14,18–20^ and to picoeukaryotes in vertical scale^18–20^.

The deep sea is characterized by being in near total darkness, with an average temperature <4 °C, a high hydrostatic pressure and low dissolved and particulate organic matter. Therefore, deep-sea picoeukaryotes are significantly different from those in the surface waters in terms of community structure^10^ and subsequently their trophic status. The Mariana Trench, begins at Sulphur Island in the north, and extends to Yapu Island in the southwest^21^, is the deepest part of any of the world’s oceans^22^. The deepest part of the Mariana Trench is located at its southern end, it is an east-west trending basin called Challenger Deep^23^, and it is both geographically and hydrotopographically isolated from other trenches in the Western Pacific^24^. Recent studies have highlighted the importance and impact of suspended organic matter on the microbial communities in bathypelagic zone and abyssopelagic zone ecosystems^25–27^. In addition, the steep slope, narrow geomorphology, and slow trench current might also help to provide a steady supply (or the occasional input) of sinking and suspended organic matter^24,28,29^, which might influence the geochemical cycle within the waters of the entire trench. To date, only prokaryotic communities in the water column^24^ and sediment^30,31^, as well as fungi in the sediment^32,33^ have been investigated in the Mariana Trench, whereas picoeukaryotes, which are a key component of the microbial ecosystem, have not been well studied.

In this new study, we investigated the diversity and activity of picoeukaryotes in the water column of the Mariana Trench, from the surface to the abyssopelagic zone and from the north to south slopes. In addition, their roles in marine microbial food webs and linkages to environmental factors were discussed in an attempt to integrate the community structure with their ecological functions.

## Results

### Hydrographic conditions

**Figure 1:**
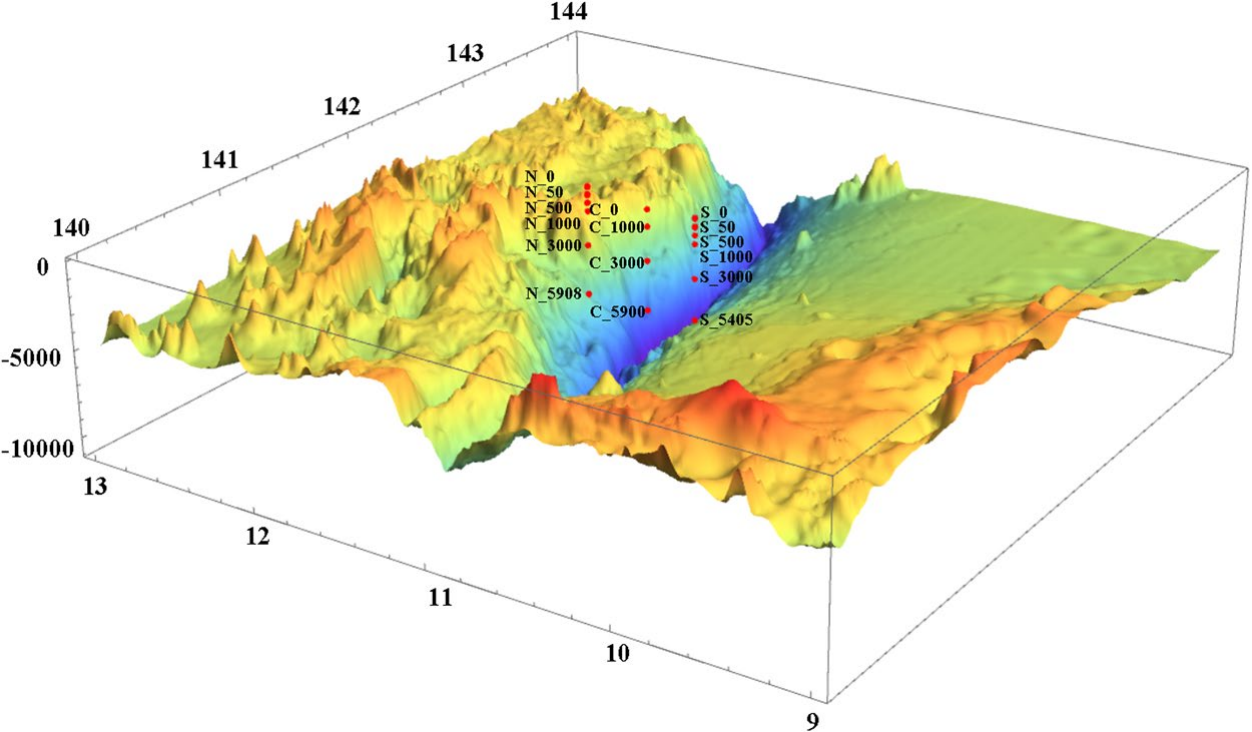
Map of the sampling stations located on the North, Center and South slopes of the Mariana Trench, where samples were collected during a cruise in 2016.

In general, similar vertical profiles of hydrographic conditions were detected for the North, Center and South stations of the Mariana Trench in the Western Pacific Ocean (Figs 1 and S1). For example, at each of the three stations, the temperature decreased sharply from ~29 °C to ~7.1 °C between depths of 50 m to 500 m, which corresponds with a stable thermocline (Fig. S1A). The salinity increased from ~34.2 psu at the surface to ~34.8 psu in the mesopelagic zone (Fig. S1B). The maximum concentrations of nitrate and phosphate were achieved in the mesopelagic zone. The concentrations of phosphate remained elevated in the deeper waters, while that of nitrate decreased sharply in bathypelagic zone at the North and South stations (Fig. S1C, S1D). The concentration of ammonia reached a maximum at a depth of around 1000 m at the North and South stations, but in the deeper water, it decreased sharply to a concentration similar to that of the surface water (Fig. S1E). However, a maximum concentration of ammonia occurred at the Center station around 4000 m (Fig. S1E). The concentration of silicate increased with depth and was thus higher in the bathypelagic zone than in the mesopelagic and epipelagic zones (Fig. S1F).

### Diversity and abundance of picoeukaryotes

**Figure 2:**
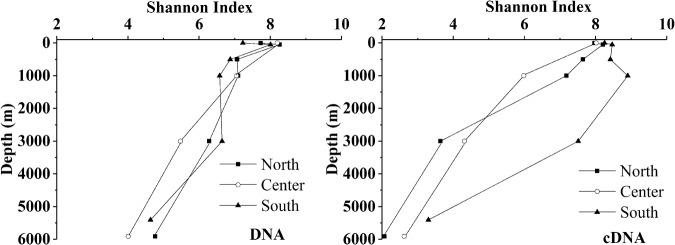
The Shannon index for the samples collected from the different depths, which are grouped according to the three stations (North, Center and South), as revealed by DNA and cDNA.

In total, 2,328,365 sequences and 5602 OTUs were generated from quality reads using 97% as clustering threshold (Table 1). Along the vertical profiles, the highest richness (Chao1) and diversity (Shannon index) of picoeukaryotes (identified at DNA and cDNA levels), occurred at a depth of 50 m (Table 1), and this pattern was also demonstrated by rarefaction curves (Fig. S2). Picoeukaryotic diversity was higher at cDNA level than DNA level, and the former exhibited more obvious variation among the three stations (Fig. 2).

**Table 1:** Sequencing information and diversity parameters of picoeukaryotes in water columns in the north, south and central regions of the Mariana Trench.

Datasets	Stations	Depth(m)	Original Reads	Quality Reads	OTUs	Shannon	Chao1	Simpson	Good’s coverage
DNA	North	0	79,116	74,746	1,281	7.73	22883.79	0.98	0.95
		50	75,019	70,975	1,877	8.26	24370.49	0.97	0.93
		500	76,014	72,435	1,460	7.07	18305.75	0.96	0.94
		1,000	83,609	79,652	1,449	7.10	11247.73	0.97	0.97
		3,000	75,653	71,907	1,114	6.28	6450.10	0.94	0.98
		5,908	66,561	62,557	1,202	4.76	8135.66	0.76	0.97
	Center	0	73,885	70,218	1,797	8.20	27469.96	0.98	0.93
		1,000	72,871	69,366	1,413	7.05	12995.06	0.97	0.96
		3,000	80,355	75,610	953	5.48	6425.77	0.92	0.98
		5,900	70,693	65,168	721	4.01	3672.18	0.77	0.99
	South	0	74,136	70,442	1,545	7.23	8825.098	0.97	0.97
		50	88,614	83,917	1,838	8.01	18484.97	0.97	0.95
		500	74,043	70,541	1,267	6.88	12435.03	0.97	0.96
		1,000	80,873	77,108	1,319	6.58	11470.12	0.94	0.97
		3,000	86,450	82,313	1,004	6.64	3433.53	0.97	0.99
		5,405	70,064	66,199	710	4.63	4195.37	0.84	0.99
cDNA	North	0	78,664	74,401	1,617	7.97	25094.39	0.98	0.94
		50	74,928	70,615	1,654	8.20	29885.04	0.98	0.92
		500	88,58	83,670	1,589	7.64	27312.72	0.94	0.93
		1,000	85,038	80,358	1,591	7.18	22283.60	0.91	0.94
		3,000	70,892	67,004	946	3.64	14565.00	0.55	0.96
		5,908	65,986	61,869	579	2.06	6092.60	0.32	0.98
	Center	0	79,509	74,095	1,628	8.02	25493.37	0.98	0.93
		1,000	80,428	75,948	1,368	5.98	18603.71	0.85	0.95
		3,000	74,841	69,959	820	4.31	10992.74	0.81	0.97
		5,900	67,148	62,726	660	2.62	7702.13	0.44	0.97
	South	0	81,739	77,267	1,652	8.25	28231.53	0.98	0.93
		50	79,192	74,614	1,791	8.47	26173.44	0.98	0.93
		500	71,392	67,207	1,502	8.42	27526.77	0.98	0.91
		1,000	77,116	72,635	1,711	8.90	32317.15	0.99	0.92
		3,000	79,029	74,589	1,348	7.51	24207.65	0.97	0.94
		5,405	82,600	78,254	825	3.30	9314.67	0.63	0.97

Note: OTUs were defined with 97% as the cutoff value and OTUs belonging to Metazoa were excluded.

**Figure 3:**
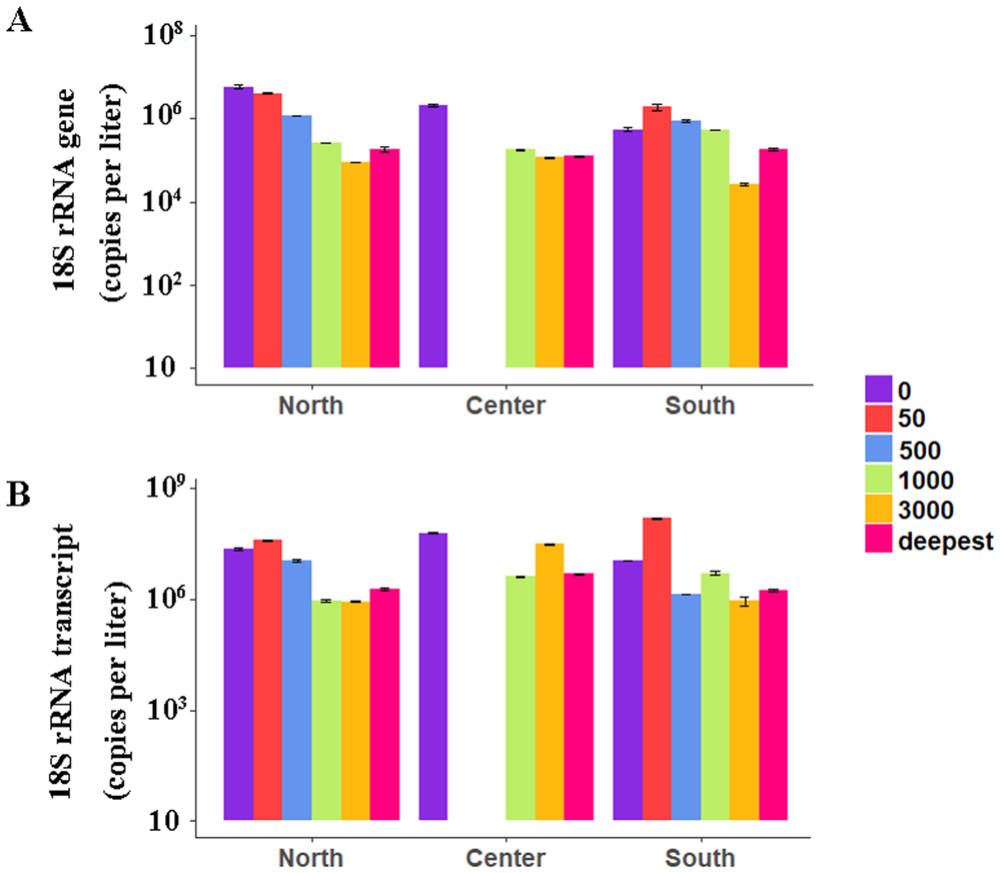
Picoeukaryotic quantification obtained by qPCR from the three stations in the Mariana Trench with (**A**) the DNA and (**B**) the cDNA datasets. ‘Deepest’ represents the deepest water at three stations; thus 5908 m, 5405 m and 5900 m at the North, South and Center stations, respectively.

The abundance of picoeukaryotic 18S rRNA gene copies and gene transcripts reached a maximum in the shallower waters (i.e., 0 m and 50 m), and then decreased in the bathypelagic zone (3000 m) at the North and South stations (Fig. 3). At the Center station, the same trend was observed for the 18S rRNA gene copies, but not at the transcript level, which showed no decrease in the deeper waters. Higher abundance of the 18S rRNA gene was detected at North station, although the abundance of the 18S rRNA gene transcript at 50 m and 1000 m was higher at South station (Fig. 3).

### Community composition of picoeukaryotes

**Figure 4:**
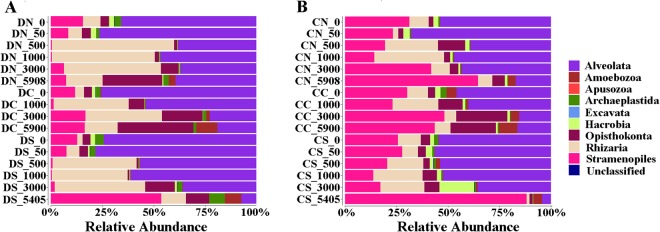
The relative abundance of the various picoeukaryotic communities in the water column at the three stations, as revealed by the (**A**) DNA and (**B**) cDNA datasets. DN, DC and DS indicate DNA at the North, Center and South stations, respectively, while CN, CC and CS indicate cDNA at the North, Center and South stations, respectively. In each case, the numbers indicate the water depth.

Four super-groups, i.e., Alveolata, Opisthokonta, Rhizaria, and Stramenopiles, dominated in both the DNA and cDNA datasets. At DNA level (Fig. 4A), the relative abundance of Alveolata decreased as the depth of the water increased. In contrast, the abundance of Opisthokonta increased with depth. The highest abundance of Rhizaria was at depths between ~500 m and ~3000 m (i.e., in the mesopelagic and bathypelagic zones), whereas that of Stramenopiles was in the surface and abyssopelagic zones. At cDNA level (Fig. 4B), somewhat similar depth-related trends were detected in the water column. Alveolata stayed relatively constant (and at a high level) from ~0 to1000 m, but then decreased with the increase of depth. Opisthokonta were found at relatively low numbers at all depths, except in the deep waters at the Center station. Rhizaria was most abundant in the mesopelagic zone at all three stations, whereas Stramenopiles was most abundant in the surface and abyssopelagic zones. Among the three stations, the highest proportion of Opisthokonta (at both DNA and cDNA levels) was observed at the Center station; whereas the highest proportion of Rhizaria and Alveolata at DNA and cDNA levels, respectively, was observed at the North station (Fig. 4).

The Alveolata super-group comprised Ciliophora, Dinophyceae, MALV-I and MALV-II, with MALV-II and Ciliophora being the dominant assemblages at DNA and cDNA levels respectively (Fig. S3A). Fungi were the main component of the Opisthokonta super-group at both DNA and cDNA level (Fig. S3B). RAD-B was the predominant group in the Rhizaria super-group, especially in the deep waters (Fig. S3C). In addition, within the Stramenopiles super-group, MAST and Bicoecea dominated in the surface and deep waters respectively, with a higher proportion (e.g., 27.7% Pelagophyceae at the South station) of known photosynthetic Stramenopiles (e.g., Pelagophyceae and Chrysophyceae) being recorded at cDNA level (Fig. S3D).

Statistical results demonstrated that there was a significant difference among the picoeukaryotic communities at the same water depth of the three stations (ANOVA, P < 0.0001). Heatmap plots were performed (using SIMPER in the DNA and cDNA datasets) to show the relative abundance of common OTUs, which would reveal which taxa were causing the differences among the picoeukaryotic communities in the deep waters of the three stations (Fig. S4). In the DNA dataset, the major taxa that caused differences in the picoeukaryotic communities between the North and South stations were Syndiniales, Fungi and Chrysophyceae-Synurophyceae (Fig. S4A). Syndiniales and Fungi were more abundant in the North station, whereas more Chrysophyceae-Synurophyceae existed in the South station. In the cDNA dataset (Fig. S4B), the major taxon that caused this difference was Chrysophyceae-Synurophyceae, which was more abundant in the South station.

All the data obtained from the different depths and stations were combined to compare the composition of picoeukaryotic communities in the epipelagic (0 m and 50 m), mesopelagic (500 m and 1000 m) and bathy- and abyssopelagic (3000 m and deeper) layers of the water column of the Mariana Trench, as revealed by the DNA and cDNA datasets (Fig. S5). The analysis of tags at the super-group level indicated that the surface was dominated by Alveolata with 74% of the total DNA reads (Fig. S5A). This was followed by Stramenopiles, Rhizaria, and Opisthokonta, with 11%, 6% and 4% of the total DNA reads, respectively. The other super-groups (e.g., Excavata, Amoebozoa, Apusozoa, Archaeplastida, and Hacrobia), collectively contributed only to ca. 5% of the total reads. When comparing the cDNA dataset with the DNA dataset, the top four groups remained in the same order, but in different proportions (Fig. S5A). The percentage of Alveolata decreased to just 56% of the total reads, whereas the share of Stramenopiles increased to 28% of the total reads. In contrast, the proportion of total reads of Rhizaria (8%) and Opisthokonta (3%) were similar in the cDNA dataset as they were in the DNA dataset. The picoeukaryote community in the mesopelagic layer was dominated by Alveolata (51%) and Stramenopiles (45%) in the DNA dataset, but Stramenopiles decreased to 25% of the total reads in the cDNA dataset. In the bathypelagic and abyssopelagic layer, the various super-groups were relatively evenly distributed in the DNA dataset, but marked differences were observed when comparing the DNA and cDNA datasets. In the latter, Stramenopiles was clearly the dominant super-group, representing over half (54%) of the total RNA reads (Fig. S5A). In terms of OTU richness, the DNA and cDNA datasets showed similar patterns in the four most dominant super-groups at the different depths (Fig. S5B). In each case, the highest number of OTUs (indicating a high diversity) was found in the Alveolata super-group.

### Relative activity of the major picoeukaryote groups

The cDNA to DNA ratios, used to indicate *in situ* picoeukaryote metabolic activity, revealed a depth-related pattern for most of the picoeukaryote groups (Table 2). The highest activity of Cercozoa (1.69), Acantharea (1.71) and RAD-B (2.93) was found in the epipelagic zone (i.e., at 0 m and 50 m), while that of Ciliophora (41.28), Choanoflagellatea (17.08), RAD-C (15.88), and Stramenopiles (e.g., Bacillariophyta (10.22), Chrysophyceae (17.43), Pelagophyceae (31.00) and MAST (10.26)), were found in the mesopelagic layer (500 m and 1000 m). Among the three stations, Ciliophora RAD-C, Chrysophyceae, Pelagophyceae, and Labyrinthulea were highly active at all three sites, but Ciliophora showed the highest level of activity of all at the North station (44.36), whereas Pelagophyceae were most active in the South station (17.82) (Table 2).

**Table 2:** The cDNA: DNA ratio of the four dominant super-groups at the taxonomic level.

		cDNA reads	DNA reads	cDNA/DNA	Average cDNA:DNA ratio			Average cDNA:DNA ratio		
					Epi-	Meso-	Bathy-; Abysso-	North	Center	South
Alveolata	Ciliophora	98,006	5,076	19.31	17.52	41.28	10.92	44.36	12.51	13.14
	Dinophyceae	79,649	50,864	1.57	1.30	5.21	0.94	1.66	0.80	2.11
	MALV-I	34,500	125,261	0.28	0.24	0.43	0.17	0.21	0.32	0.31
	MALV-II	31,179	241,305	0.13	0.14	0.13	0.12	0.11	0.11	0.15
Opisthokonta	Choanoflagellatea	3,124	1,186	2.63	2.03	17.08	3.13	2.12	3.20	2.89
	Fungi	30,632	58,265	0.53	0.48	2.01	0.26	0.54	0.52	0.52
Rhizaria	Cercozoa	8,212	29,641	0.28	1.69	0.21	0.21	0.29	0.22	0.29
	Acantharea	27,188	115,467	0.24	1.71	0.13	0.03	0.19	0.27	0.28
	Polycystinea	3,359	23,484	0.14	0.03	0.18	0.09	0.05	0.71	0.10
	RAD-B	37,381	49,599	0.75	2.93	1.37	0.30	0.85	0.46	0.82
	RAD-C	5,841	584	10.00	7.43	15.88	4.21	12.01	4.39	12.84
Stramenoplies	Bacillariophyta	788	569	1.38	1.20	10.22	0.74	1.55	1.58	1.12
	Chrysophyceae	84,535	33,820	2.50	4.21	17.43	2.03	6.75	4.76	2.04
	Dictyochophyceae	4,370	3,639	1.20	1.00	7.33	3.16	0.74	2.60	1.68
	Pelagophyceae	9,235	778	11.87	12.43	31.00	5.58	7.11	12.97	17.82
	Bicoecea	19,234	11,453	1.68	7.36	14.21	1.06	1.36	1.32	6.48
	Labyrinthulea	14,170	2,003	7.07	3.04	21.97	2.58	11.00	2.09	10.16
	MAST	48,685	22,429	2.17	1.68	10.26	3.38	2.25	1.89	2.23
	MOCH	6,619	2,653	2.49	2.23	11.28	2.34	2.56	3.74	2.13

Note: Epi-: epipelagic zone; Meso-: mesopelagic zone; Bathy- and Abysso-: bathypelagic and abyssopelagic zones.

### Community comparison and environmental impacts

**Figure 5:**
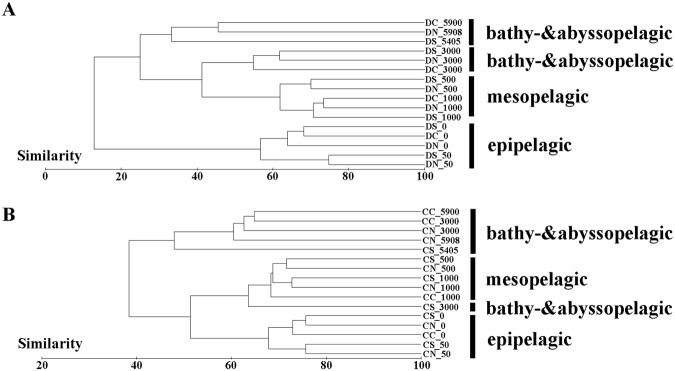
UPGMA cluster diagram of the Bray-Curtis similarities calculated from square-root transformed relative OTU abundances for the (**A**) DNA and (**B**) cDNA datasets.

**Figure 6:**
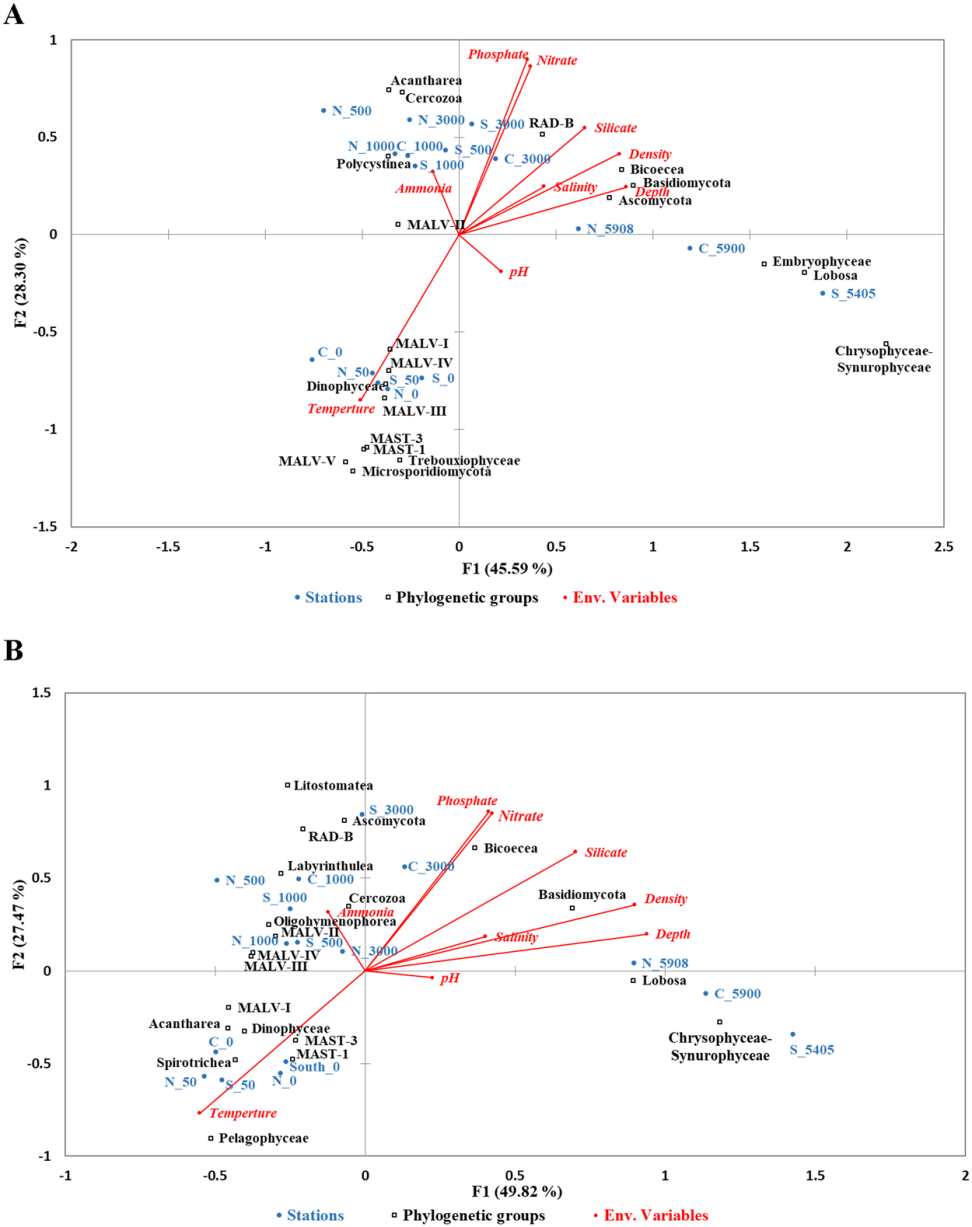
Correspondence canonical analysis (CCA) biplots showing the variable composition of picoeukaryotes in relation to important environmental factors at the three stations in the Mariana Trench in the (**A**) DNA and (**B**) cDNA datasets.

Significant differences in the composition of the picoeukaryotic communities were observed when comparing the DNA and cDNA results (ANOVA, p < 0.0001) and the surface and deeper waters (ANOVA, p < 0.0001). This finding was supported by the UPGMA clustering, which demonstrated that samples from the surface (i.e., 0 m and 50 m) were closely clustered together but clearly separated from the samples collected in the deeper waters (i.e., >500 m) at both DNA (Fig. 5A) and cDNA (Fig. 5B) levels. In addition, a CCA analysis demonstrated that ~74% (DNA dataset; Fig. 6A) and ~77% of the variance (cDNA dataset; Fig. 6B) could be explained by the first two axes. A separation of taxonomic compositions between samples from the shallow waters and deeper waters (>500 m) was also observed, determined by water depth-associated parameters, such as phosphate, nitrate and temperature. Low phosphate, nitrate and high temperature in the shallow waters were important factors for this clustering, which were highly correlated with sampling depths. In the DNA dataset, MALV-I, MALV-III, MALV-IV and Dinophyceae frequently occurred in shallow water. RAD-B appeared to be associated with high silicate, while MALV-II showed a minimal relationship with the variables being tested. In cDNA dataset, MALV-I, MAST-1, MAST-3, Sprotrihea and Dinophyceae frequently occurred in shallow water, Bicoecea appeared to be associated with high phosphate and nitrate.

## Discussion

So far it has been estimated that only 5% of the deep ocean has been explored in detail, and that less than 0.001% has been sampled and described in terms of biodiversity^34,35^, and even less is known about the picoeukaryotes that reside there. In this study, the composition of picoeukaryotes communities residing in three geographically separate marine water columns (from the surface to the abyssopelagic zone) in the Mariana Trench were investigated.

The widespread distribution of the major picoeukaryotic groups (e.g., Ciliophora, MALV-I, MALV-II and Radiolaria) at different depths of the Mariana Trench confirmed their diverse range of habitats. Alveolata display a high level of diversity, which might contribute to their successful colonization over a wide range of ecological niches. Ciliophora are already well known to be the major grazers of bacteria and pico-/nano-sized eukaryotes in the epipelagic zone^3,14^. In our study, they were found to be active throughout the whole water column of the Mariana Trench, even in the abyssopelagic zone. This phenomenon has also been observed in the South China Sea^20^. Consistent with the results acquired from a previous global ocean survey^36^, MALV-I and MALV-II were also shown to be dominant in all the samples obtained from the Mariana Trench. In addition, it is well known that Radiolaria are one of the major players that export organic carbon to the deep sea^37,38^, and that they also host many other living microorganisms, such as Prymnesiophyceae, Prasinophyceae, and dinoflagellates^39^. Ciliophora and Radiolaria-affiliated sequences detected in this study could be derived from small fragments of these organisms that passed through the 3 µm filters^40,41^. The high diversity of Radiolaria recorded in our study might suggest that they also play a crucial role in helping to maintain the microbial ecosystems of the Mariana Trench. MAST-3, the main heterotrophic taxon in Stramenopiles, was frequently observed across the water column, whereas representative MAST-1 clades (e.g., C and D) and MAST-4 clades (e.g., A, C, D and E), key heterotrophic predators both in coastal waters and open ocean^2,3^, were mainly detected in the surface waters. This might be due to the relatively abundant food source in the surface layer of the Mariana Trench for these two clades. Prymnesiophyceae is known to be an important primary producer in the open ocean^42^, which exists at high diversity^43^, but usually low abundance^44–47^. A low proportion of Prymnesiophyceae present throughout the whole water column in our study may be caused by the biased primers applied^48,49^.

Our extensive sequencing effort provides a more comprehensive molecular description of the diversity of marine picoeukaryotes in the Mariana Trench. Since extracellular RNA is much less stable and therefore has a much shorter lifetime than DNA, the bias of taxon-specific rDNA copy number can be avoided by analyzing cDNA from extracted RNA^19^. In our study, MALV-III, Ciliophora and RAD-C were either at low abundance or else not detected at all in the DNA dataset, but they contributed notably to the cDNA dataset (Fig. S3). This might indicate that these taxa have a relatively higher metabolic activity at the time of sampling, or else that they have fewer rDNA copies. In contrast, MALV-I and MALV-II were the dominant picoeukaryotes in the DNA dataset but they contributed little to the cDNA dataset. This phenomenon has also been reported previously for MALV^3,50^, and it might reflect the high 18S rDNA gene diversity and higher genomic copy number observed, which matches their parasitic life strategy, such that they are relatively inactive and possess fewer ribosomes than other picoeukaryotes^51,52^.

The presence of a large gradient of physico-chemical conditions along the water depth, and the difference in geographical location are both important factors that might influence the composition of microbial communities^53,54^. In our study, a vertical distribution pattern along the water depth was found, characterized by the predominance of Alveolata, Rhizaria and Opisthokonta in the bathypelagic zone. This is in agreement with the findings of the Malaspina expedition, which reported that Alveolata, Rhizaria and Fungi were the dominant groups in the bathypelagic waters of Atlantic, Pacific and Indian Oceans^13^. Similar picoeukaryotic compositions in the bathypelagic zones may be an adaptation to the deep-sea conditions. Deep-sea assemblages of protist have recently been proposed^37^, and our study has expanded the deep-sea assemblages deeper to the abyssopelagic zone as revealed by the UPGMA clustering. Some studies have revealed that deep-sea biogeochemical cycles are more complex than previously expected, and there is a mismatch between the organic carbon supply and the microbial heterotrophic demand^24,55,56^. This could be attributed to mixotrophy, whose contribution to the biogeochemical cycle in the dark ocean may be remarkable^24^.

Our picoeukaryotes abundance ranged 10^3^~10^4^ cell/ml, which is of the same order of magnitude with data reported in the surface waters^57^. We did find a decrease of picoeukaryotes abundance with increase of water depths, however, since we are the first to report the picoeukaryotic abundance at the bathypelagic and abyssopelagic zones based on 18S rRNA gene, the deep sea picoeukaryote abundance needs be confirmed by more studies. In addition, the abundance of gene transcript was generally higher than that of gene abundance for active picoeukaryotes^58^. The relative higher abundance of gene transcript than gene found in our study suggested that the picoeukaryotic groups in the Mariana Trench were metabolically active.

The cDNA: DNA ratio has been previously employed as a proxy of relative metabolic activity^14,20,36^. However, it should be pointed out that the cell size^59^ might limit the use of the cDNA: DNA ratio to evaluate microbial activity, which can also be influenced by life histories, life strategies and non-growth activities among species^14,60^. Therefore, it is prudent to compare the cDNA: DNA ratios among related species that have comparable cell sizes or metabolisms. In this case, the bias of taxon-specific rDNA copy number is better avoided by analyzing cDNA from extracted RNA. In our study, determination of average cDNA: DNA ratios revealed several vertical trends as well as a few horizontal spatial trends. Most picoeukaryote groups showed a depth-related distribution pattern for their relative activities (Table 2). The fact that the highest activity of most groups was found in the mesopelagic zone (i.e., at 500 m and 1000 m) might be caused by the active predation on prokaryotes by some groups of small eukaryotes such as heterotrophic flagellates in this transition zone^25^. For example, the higher relative activity of MASTs (10.26), which are known to be abundant bacterial grazers^61^ in the mesopelagic zone, together with the widespread distribution of these taxa, might reflect their active grazing activities on prokaryotes in Mariana Trench. Variations in the metabolic activity of picoeukaryotic assemblages among the three stations were also identified in our study. RNA: DNA ratios for most groups in the Alveolata (e.g., Dinophyceae and Ciliophora), Rhizaria (e.g., RAD-C) and Stramenopiles (e.g., Chrysophyceae, Pelagophyceae and MASTs), were consistently higher at the North or South stations, when compared with the Center station. It is obvious that the steep slopes and narrow geomorphology supplying the occasional input of sinking and suspended organic matter^24^ might also influence the picoeukaryotic activity in the Mariana Trench. However, the spatial profiles revealed in our present study represents only a tiny fraction of the situation in the global oceans. A more comprehensive survey over a larger geographical scale is required in order to define the functions of picoeukaryotes in marine biogeochemical cycling and their responses to environmental change, on a global scale.

The presence of well-preserved phytoplankton cells in the deep sea have been reported based on direct microscopy for a long time^62,63^. A global study on the diversity of bathypelagic microbial eukaryotes based on DNA sources showed the presence of photoautotrophic groups in the deep sea, e.g. Bacillariophyta, Dictyochophyta, Prasinophyceae, Prymnesiophyceae, and Raphidophyta^64^. Moreover, study in the South China Sea also revealed that photoautotrophic groups accounted for ca. 0.9–4.3% of the total RNA reads which is even higher than their contribution in the DNA dataset^20^. In present study, after removing the OTUs only represented in RNA or DNA datasets, the photoautotrophic groups were still present (Fig. S3) and having even higher metabolic activity in the mesopelagic zone (Table 2). Fast-sinking has been considered as the main reason for the presence of photoautotrophic cells in the dark ocean^65^. However, the organisms we studied were within the pico-sized fraction, it will take long time for them to sink to the deep sea, and the possibility still being metabolically active was very low. On the other hand, the mixotrophic life style could be a potential reason, because mixotrophy was reported as the dominant life style for many mesopelagic active photosynthetic groups^7,20,66,67^.

Recent studies have revealed that the proportion of purely autotrophic eukaryotic microorganisms in nature is lower than expected^7,68^. Many photoautotrophic picoeukaryotes are facultative heterotrophs, so they are both primary producers and consumers^20^. Mixotrophic picoeukaryotes contribute significantly to biogeochemical cycles in the oceans globally^7,66,67^. Indeed, our data showed that ~49% of Stramenopilies at the deepest layer of three stations were Chrysophyceae-Synurophyceae. These two so-called ‘Golden Algae’ groups have been well documented as being photosynthetic microalgae, because some of the best-known species contain chlorophyll and fucoxanthin^69^. In fact, these two groups are ecologically important autotrophic, mixotrophic and heterotrophic flagellates and they have important functions both as primary producers and consumers of bacteria in the aquatic food chain^70^. Furthermore, Ciliophora, having the highest relative activity of the Alveolata, have long been known to be major grazers of bacteria and pico-/nano-sized eukaryotes in both productive^71^ and oligotrophic^72^ waters. Some ciliates can switch from heterotrophic to phototrophic mode by utilizing ingested chloroplasts, and in this way, they remain active under both light and dark conditions^73,74^.

In our study, Syndiniales (mainly MALV-I and MALV-II) dominated the Alveolata assemblages (Fig. S3A). Based on the life style of several species^53,75,76^, Syndiniales are believed to be parasitic and they infect a wide variety of free-living organisms, such as dinoflagellates, ciliates and Radiolaria. Syndiniales (mainly MALV-II) and Radiolaria remarkably exhibited the same trend in relative abundance along the depth, which suggests the possibility of a parasitic relationship. Recently, the flux of neutrally buoyant or slow-sinking marine snow (i.e., particulate organic matter, POM) from the epipelagic zone was thought to be an important source of nutrients for heterotrophic picoeukaryotes in the deep-sea ecosystem^26^, where the marine snow might provide hotspots of microbial diversity and activity^77^, and harbor microbes of different trophic status, such as saprotrophs, heterotrophs and parasites^7^. A recent study has shown that two saprotrophic groups (i.e., Fungi and Labyrinthulea), dominate the bathypelagic marine snow biomass^78^, indicating that eukaryotic microbes might contribute to particle solubilization and remineralization^64^. Previous surveys have reported that Fungi are dominant in the deep-sea sediment^32,33^, and in the bathypelagic zone^79,80^, as well as in several specific deep-sea environments, such as hydrothermal vents and methane cold seeps^81^. In agreement with these previous reports^79,80^, we showed that in the Mariana Trench, Fungi were dominant in the bathypelagic zone, and they were even found in the abyssopelagic zone. It has previously been reported that the marine osmoheterotrophic protist, Labyrinthulea, is a dominant and active group of Stramenopiles in the mesopelagic zone^82^. However, Thraustochytriaceae, which are affiliated to Labyrinthulea, and are a family containing parasitic or symbiotic species^83^, were more abundant in the bathypelagic environment down to 4000 m^84^ and are extremely well adapted to cold temperatures and high pressures^85^. Likewise, in our study, Thraustochytriaceae was recovered and shown to be active in the bathypelagic zone.

In summary, our study revealed general spatial distribution patterns of picoeukaryotes in the north and south slopes and center of the Mariana Trench with water depths ranging from the surface to the abyssopelagic zone. We found that four super-groups (i.e., Alveolata, Opisthokonta, Rhizaria and Stramenopiles) predominated through the water column at both DNA and cDNA levels, expanding the deep-sea assemblages from current bathypelagic to abyssopelagic zones. In addition, higher metabolic activity found at the slope stations than the central water column could be due to the higher input of sinking and suspended organic matter along the slopes. Physicochemical gradients along the water depths explained more of the picoeukaryotes population variations, since no significant difference was found among the three horizontal stations. Overall, our study provides a better understanding of the composition, diversity and metabolic activity of picoeukaryotic community in the Mariana Trench. In the future study, metagenomic approach together with sampling over a larger geographical scale will help to obtain an exhaustive picture of marine picoeukaryotes in Mariana Trench.

## Materials and Methods

### Sample collection

Samples were collected from the North (11°33′N, 142°00′E), Center (11°11′N, 141°59′E) and South (10°51′N, 141°57′E) slopes of the Mariana Trench in the Western Pacific Ocean during a cruise in June 2016 (Fig. 1). Niskin bottles were used to collect waters samples at six discrete depths at the North (0 m, 50 m, 500 m, 1000 m, 3000 m and 5908 m), and South (0 m, 50 m, 500 m, 1000 m, 3000 m and 5405 m) stations; and at four discrete depths at the Center station (0 m, 1000 m, 3000 m and 5900 m). *In situ* hydrographical parameters (i.e., temperature and salinity) were recorded at each station with a conductivity-temperature-depth (CTD) rosette system (Sea-Bird Electronics). In addition, the concentrations of nutrients (i.e., nitrate, silicate, ammonia, and phosphate) were analyzed with an auto-analyzer (QuAAtro, BLTEC. Co. Ltd.).

For DNA/RNA sample collection, about 2 L of the water samples were sequentially filtered through 3 µm to 0.22 µm pore size polycarbonate filters (47 mm, EMD Millipore, Billerica, MA, USA). Filters for RNA extraction were immediately immersed in RNA later solution (Ambion) to avoid RNA degradation. All filters were flash frozen and stored at −80 °C until further analysis.

### Nucleic acid extraction, PCR amplification and sequencing

Total DNA was extracted from the 0.22 µm polycarbonate filters with a PureLink Genomic DNA kit (Invitrogen, Carlsbad, CA), following the manufacturer’s instructions. Total RNA was extracted with TRIzol® Reagent and RNA purification kits (Invitrogen, Carlsbad, CA). The concentrations of total DNA and RNA acquired, were quantified with a NanoDrop 2000 Spectrophotometer (Thermo Scientific, Thermo Fisher Scientific, Corp.) and the quality was checked via gel electrophoresis. The total RNA was purified with DNase I (Ambion, Life Technologies, USA) to eliminate DNA contamination, after which efficient digestion was confirmed by gel electrophoresis. The purified RNA was then reverse transcribed to cDNA using the Superscript III First-strand Synthesis System for RT-PCR kit (Invitrogen, Carlsbad, CA). The synthesized cDNA was then digested with 2U RNase H at 37 °C for 20 min to remove RNA residue and it was then used for subsequent PCR amplification. Both DNA and cDNA were amplified using the FastStart High Fidelity PCR system (Roche) with the following universal primers: TAReuk454FWD1 (5′-CCAGCA(G⁄C)C(C⁄T)GCGGTAATTCC-3′) and REV3 (5′-ACTTTCGTTCTTGAT(C⁄T)(A⁄G)A-3′)^86^, to target the V4 domains of the 18S rRNA gene. The polymerase chain reaction was performed with an initial denaturation step of 95 °C for 3 min, followed by 32 cycles of: 95 °C for 30 s, 55 °C for 30 s and 72 °C for 1 min, after which there was a final extension step of 72 °C for 5 min. A negative control of double-distilled water was also performed during amplification in order to avoid reagent contamination. Amplification and paired-end sequencing of the amplicons were performed with an Illumina HiSeq PE250 sequencer (Novogene Co., Ltd., www.novogene.com).

### Quantitative PCR

The abundance of the 18S rRNA gene and gene transcript were quantified using the StepOnePlus quantitative PCR (qPCR) system (Applied Biosystems Inc., Carlsbad, CA, USA). Each qPCR reaction comprised 10 μL 2 × SYBR® Premix Ex Taq™ II (Takara Bio Inc., Shiga, Japan), 0.3 μM euk345f/Euk499r primer^59^, 2 μL DNA/cDNA as the template, 0.4 μL ROX reference dye, and water to a total of 20 μL. Quantitative PCR reactions and calibrations were performed according to Zhu’s paper^59^. Triplicate qPCR reactions were performed for each sample with efficiencies of ~102%, and the gene copy number was normalized to the quantity of the gene and gene transcripts.

### Bioinformatics analysis

After sequencing, overlapping reads were merged and barcoded, and low quality sequences were removed using Qiime with default parameters^87^. Chimeras were detected and removed with UCHIME against PR^2^ database^88^, and reads presented as a single copy (singleton) were removed too. The remaining reads were then clustered into Operational Taxonomic Units (OTUs) at 97% sequence similarity. Taxonomy assignment of OTUs that were not affiliated with picoeukaryotes (including bacteria and archaea, as well as metazoan and plastidial sequences), as determined from the PR^2^ database^88^, were further removed^89^. A filtered OTUs table of each sample was generated with QIIME 1.9.1. Diversity estimations (rarefaction analyses, Chao1, Shannon, Simpson and Coverage) were calculated using QIIME^87^.

### Statistical analysis

The unweighted pair group method with arithmetic mean (UPGMA) was used to study the distribution pattern of picoeukaryotic communities based on the Bray-Curtis similarity index within PRIMER 5 (Plymouth Marine Laboratory, Plymouth, UK)^90^. Visualization of community structure was conducted using the R software (R version 3.3.3). Canonical correspondence analysis (CCA) was performed using XLSTAT to identify a possible differentiation of the communities under the constraint of environmental factors, and assess correlations between environmental variables and community variability. One-way analysis of variance (ANOVA) and similarity percentage analysis (SIMPER) were conducted in Paleontological Statistics (PAST) version 3^91^ using relative abundance of OTUs to detect whether the difference between two or more samples is statistically significant.

## Electronic supplementary material

Supplementary Figures

## Data Availability

All of the 18S rRNA gene and gene transcript sequences obtained from this study have been deposited in the National Center for Biotechnology Information (NCBI) Sequence Read Archive (SRA) under the accession number SRP141405.
