# Supplementary material for: Spatial Variability of Picoeukaryotic Communities in the Mariana Trench
Source: Sci Rep. 2018 Oct 18;8:15357. doi: 10.1038/s41598-018-33790-4 (PMC6194128; doi:10.1038/s41598-018-33790-4)
Supplement: Supplementary file 1 — Supplementary Figures [file 41598_2018_33790_MOESM1_ESM.doc]

**Spatial Variability of Picoeukaryotic Communities in the Mariana Trench**

**Hongmei Jing^1^*, Yue Zhang^1, 2^, Yingdong Li^3^, Wenda Zhu^1, 2^, Hongbin Liu^3^***

*^1^CAS Key Laboratory for Experimental Study under Deep-sea Extreme Conditions, Institute of Deep-sea Science and Engineering, Chinese Academy of Sciences, Sanya, China*

*^2^University of Chinese Academy of Sciences, Beijing, China*

*^3^Division of Life Science,* *The Hong Kong University of Science and Technology, Clear Water Bay, Kowloon, Hong Kong SAR, China*

**Supplementary Figures**

**

**

**Figure S1.** Vertical profile of the hydrographical conditions of the sampling stations in the Mariana Trench, as follows: (A) temperature; (B) salinity; (C) nitrate; (D) phosphate; (E) ammonia; (F) silicate.


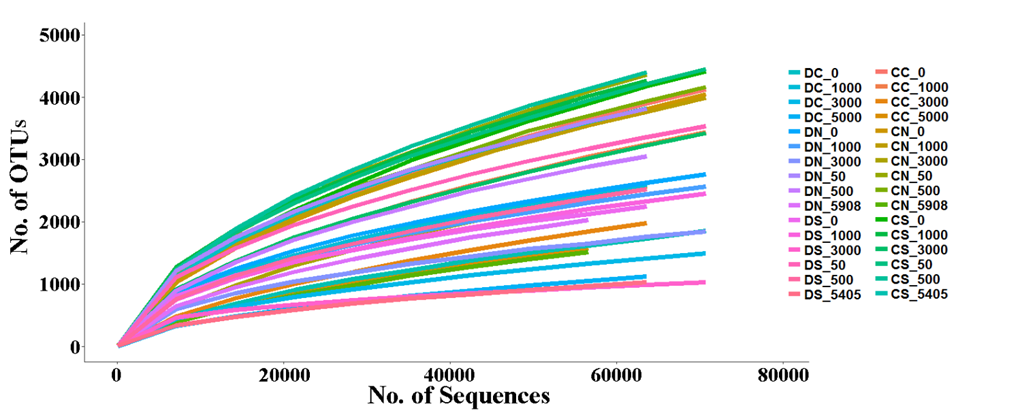


**Figure S2.** Rarefaction curves of each sample's DNA and cDNA sequencing at 97% threshold


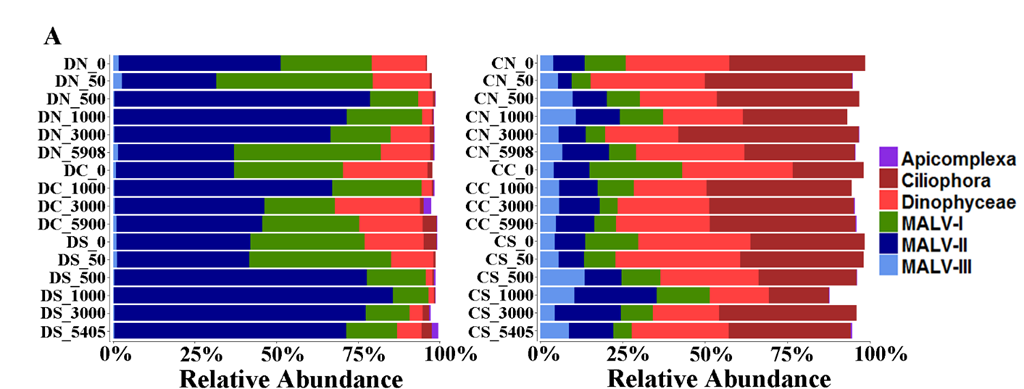


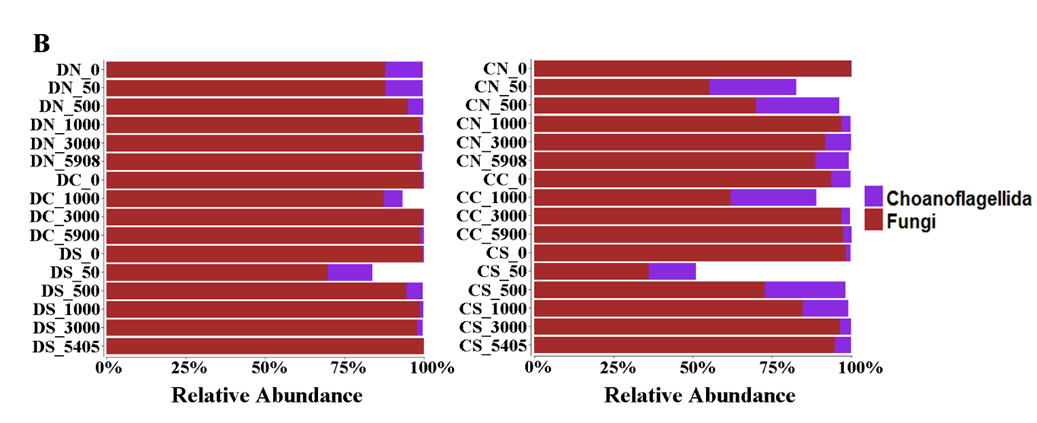


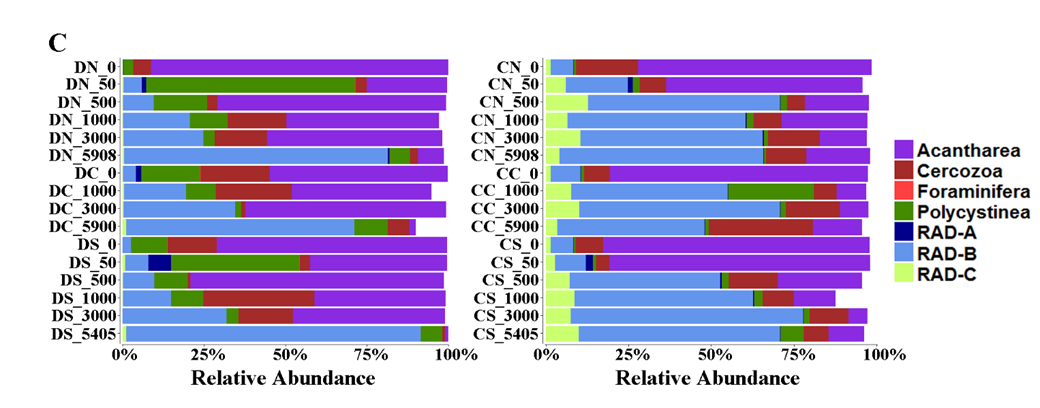


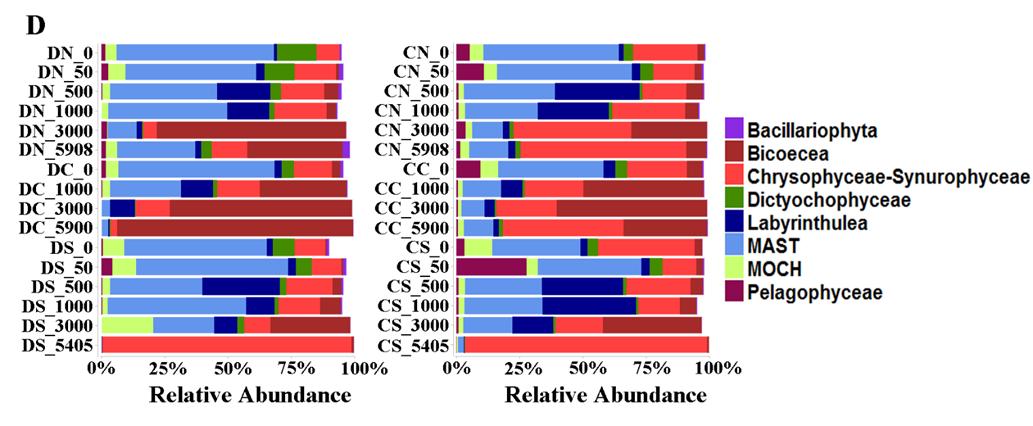


**Figure S3.** Relative abundance of the major picoeukaryotes at the 4 dominant super-group taxonomic level, (A) Alveolata, (B) Opisthokonta, (C) Rhizaria, and (D) Stramenopilies, as revealed by the DNA and cDNA datasets, respectively.


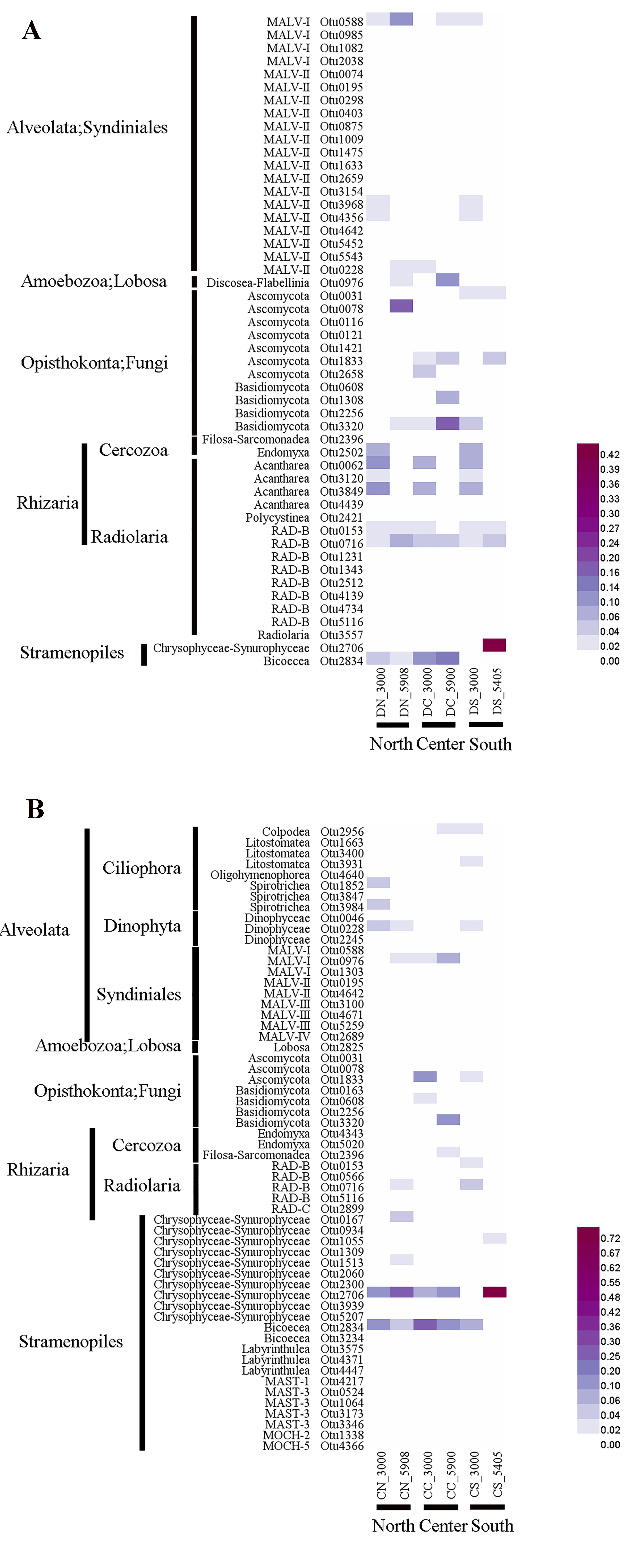


**Figure S4.** Heatmap plots showing the relative abundance of common OTUs, which might explain the differences observed between the three stations in the picoeukaryotic communities in the deep ocean, in the (A) DNA and (B) cDNA datasets.


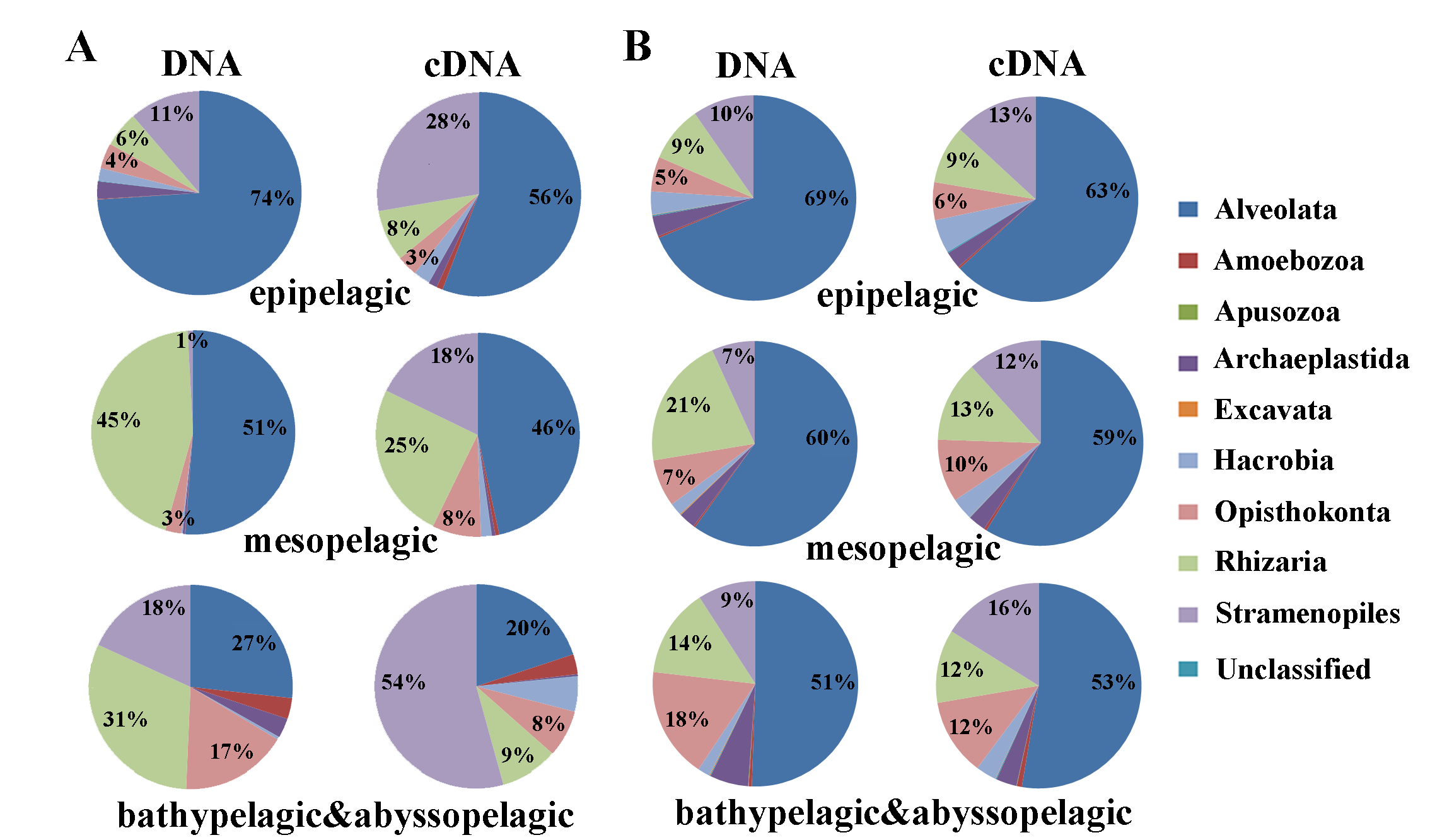


**Figure S5.** Overview of the diversity of picoeukaryotes at the super-group taxonomic level. (A) Number of tags per super-group, averaging the relative abundance at different depths. (B) Number of OTUs clustered at 97% similarity per super-group.
